# Partial proteolysis improves the identification of the extracellular segments of transmembrane proteins by surface biotinylation

**DOI:** 10.1038/s41598-020-65831-2

**Published:** 2020-06-01

**Authors:** Tamás Langó, Zoltán Gergő Pataki, Lilla Turiák, András Ács, Julia Kornélia Varga, György Várady, Nóra Kucsma, László Drahos, Gábor E. Tusnády

**Affiliations:** 1 Institute of Enzymology, Research Centre for Natural Sciences, Magyar tudósok krt 2, Budapest, H-1117 Hungary; 2 Present Address: Soft Flow Ltd. Ürögi fasor 2/a, Pécs, H-7634 Hungary; 3 Institute of Organic Chemistry, Research Centre for Natural Sciences, Magyar tudósok krt 2, Budapest, H-1117 Hungary

**Keywords:** Bioinformatics, Proteomic analysis, Molecular modelling, Membrane proteins

## Abstract

Transmembrane proteins (TMP) play a crucial role in several physiological processes. Despite their importance and diversity, only a few TMP structures have been determined by high-resolution protein structure characterization methods so far. Due to the low number of determined TMP structures, the parallel development of various bioinformatics and experimental methods was necessary for their topological characterization. The combination of these methods is a powerful approach in the determination of TMP topology as in the Constrained Consensus TOPology prediction. To support the prediction, we previously developed a high-throughput topology characterization method based on primary amino group-labelling that is still limited in identifying all TMPs and their extracellular segments on the surface of a particular cell type. In order to generate more topology information, a new step, a partial proteolysis of the cell surface has been introduced to our method. This step results in new primary amino groups in the proteins that can be biotinylated with a membrane-impermeable agent while the cells still remain intact. Pre-digestion also promotes the emergence of modified peptides that are more suitable for MS/MS analysis. The modified sites can be utilized as extracellular constraints in topology predictions and may contribute to the refined topology of these proteins.

## Introduction

In the living organism, all cells and their organelles are separated by lipid bilayers from the outside environment. These biological membranes contain polypeptide chains, called transmembrane proteins (TMPs) that span the membrane one or more times. TMPs are involved in the flow of material, energy and information across the cell and organelle membranes. About 55% of the drugs currently approved by the Food and Drug Administration (FDA) target TMPs proving the importance of this class of proteins^1^. Analysis of the complete genome sequences shows that approximately 20–30% of the open reading frames encodes TMPs^2–4^. Based on the secondary structure of their transmembrane segments, they can be categorized either as α-helical or β-barrel TMPs. According to the currently available protein structural databases, more than 4300 α-helical and 450 β-barrel TMP structures have been determined so far, however, they only make up 2% of all known three-dimensional protein structures^5–7^. The traditional techniques such as NMR and X-ray crystallography are difficult to apply to TMPs due to the specific physical-chemical properties of these proteins, making these techniques time–consuming and costly. Deepening our knowledge of their structure is crucial to the development of new and effective drugs.

Although methods of high-resolution structural determination are constantly evolving, bioinformatics^3,8,9^ and other experimental methods^10,11^ have a great importance in the characterization of topology of TMPs. Topology defines the number and location of transmembrane segments (TMSs) along the protein sequence, as well as the orientation of the connecting loops relative to the membrane.

Topology prediction methods^3,9,12–14^ have been available for a long time by developing more and more accurate applications. The state-of-the-art algorithms take into account the consensus of other methods (CCTOP^15^, TOPCONS^16^) and the best ones also consider already known structural information about TMPs generated by various experimental techniques^15^.

Experimental topology data about TMPs is limited to a few thousand proteins, moreover this information is often scattered in the literature. There are various experimental techniques which can produce topology data for TMPs. These methods can be classified based on whether the coding sequence of the protein of interest (POI) is modified or not. The size of modifications on POIs is highly variable, e.g. in some cases the sequence of the target protein is genetically fused with the coding sequence of the reporter protein (such as GFP^17,18^ and PhoA fusion^19–21^). In other cases, only an individual amino acid in the POI is modified (a single cysteine^11,22,23^ or lysine^24^ residue introduced in the appropriate part of the sequence). As for protein fusions, the activity or fluorescence of the inserted protein can indicate the site of the fusion relative to the membrane. Amino acid mutagenesis gives structural information on a single position by modifying the particular residue using membrane- permeable and impermeable residue-specific chemical agents. Other approaches are N-glycosylation motif^25,26^ (NXS/T where X can be any amino acid except proline) and epitope^27–30^ insertion techniques, often employed for gathering topology data. N-glycosylation can only occur on the extra-cytosolic side of the membrane, and epitopes can be detected by specific antibodies, with or without permeabilization of the cell membrane. The main disadvantage of these methods is that the modifications sometimes affect the location or function of the target protein, making the results ambiguous and unreliable^25,27,29^.

Therefore, many researchers select those methods which examine the structure of native proteins. For example, an antibody against an endogenous epitope^31^ can provide topology data of that particular region or targeted^23,32^ and proteome-wide extracellular glycosylation site identifications^33,34^ (the latter sometimes can be ambiguously determined because asparagines can undergo spontaneous deamidation which results in a mass shift of +0.984 Da that is a similar modification after treating the glycopeptides with PNGaseF^35,36^). Mapping of the protease-accessible cleavage sites of the proteins^37^ and chemical modification of each amino acid^38,39^ can also be classified here. These procedures are often time-consuming and the interpretation of the results is often not straightforward and usually limited to the characterization of only one protein with limited topology data.

There are databases that collect the above described data, i.e. ExTopoDB^40^ and TOPDB^41,42^, the latter currently providing the most comprehensive dataset, thus it is a useful benchmark for the results of the newly developed techniques.

Detecting chemical modifications on the reactive side chains of the accessible residues with mass spectrometry provides the opportunity to determine topology data for more TMPs at the same time. Our group has already developed two residue-specific labelling techniques with membrane-impermeable agents. First, we used Sulfo-NHS-SS-biotin for modifying the side chains of lysine residues on the cell surface of red blood cells, HL60 and K562 cells. These experiments resulted in 730 extra-cytosolic labelled positions for almost 200 TM proteins^43^. Second, we targeted the carboxyl groups with a two-step reaction, first activating them with EDC and Sulfo-NHS, then labelling the activated carboxyl groups with biotinyl cystamine. This labelling method resulted in 135 new topological positions for 38 TMPs in HL60 cell line^44^.

Compared to previously existing techniques, the labelling methods proposed by us provide topology data for TMPs accurately and within a relatively short time. Despite these methods being more cost effective and quick, they also have some disadvantages. First of all, not every protein can be labelled on the cell surface, due to the lack or inaccessibility of carboxyl and primary amino groups in the extracellular domains, limiting the number of TMPs that can be characterized^43,44^. Additionally, we can only get topology data for those TMPs that are the most abundant and/or have a big extracellular domain with many targetable residues on the cell surface in these experiments. The amino group-labelling procedure has a serious limitation, because it utilizes trypsin, the most widely used protease for mass spectrometry, which cannot recognize modified side chains of lysine residues^45^. The missed cleavages lead to longer and more complex peptides, making sequencing and identification of the exact site(s) of their modification(s) in the proteomic experiments more difficult or even impossible.

The above described experimental techniques provided many, but still limited amount of topology information. To overcome this and to produce experimental data for some of the uncharacterized proteins development of new experimental techniques remain vital in promoting our understanding of TMP topologies.

**Figure 1:**
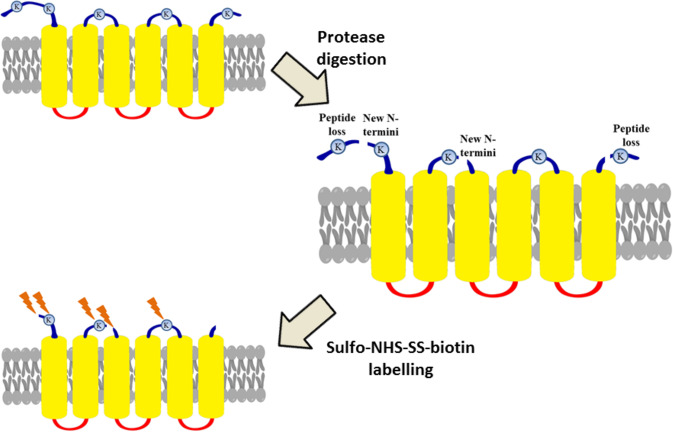
Protease treatment of TMPs to reveal new extracellular segments that can be labelled by biotinylation agents. Representation of extracellular Sulfo-NHS-SS-biotin labelling on a pre-digested model TMP (the colours of TMP based on topology: yellow: transmembrane segments, blue: extracellular parts and red: intracellular parts, orange lightning: Sulfo-NHS-SS-biotin and grey: phospholipid bilayer). After pre-digestion, new N-termini and accessible lysine (K) residues arise and can be labelled with Sulfo-NHS-SS-biotin.

The aim of this study was to develop an alternative Sulfo-NHS-SS-biotin labelling method and to partly avoid the above mentioned disadvantages to gain further experimental topology data. Here, we modified our previous protocol^43^ by introducing mild protease digestion before biotinylating the cell surface. This part of our protocol is similar to the digestion of the cell surface or the membrane shaving techniques^46,47^ where the membrane surface-exposed peptides are analysed with tandem mass spectrometry after the various protease treatment (such as trypsin, proteinase-K, thermolysin) in different cell types. These methods focus on the released peptides, that may be a value in the design of novel vaccines^46^ and in the topology prediction of TMPs^47^, but the samples often contaminated by cytosolic proteins due to the cell lysis, which can impair the detection of those peptides that originated from TMPs. In the present work, we brought those protein segments into focus that remained on the cell surface after the partial proteolysis step and could be biotinylated. In detail, we carefully optimized the digestion conditions, preserving the membrane integrity of the cells (reducing the risk of cytosolic contamination) using two different serine proteases (trypsin and chymotrypsin) separately in our experiments. While the digestion of the cell surface may lose lysines within large extracellular domains, in return for the pre-digestion step new N- and new C- termini around the cleavage sites and some earlier inaccessible lysine residues of the TMP may become accessible for labelling (Fig. 1). In addition, the method can produce modified peptides more suitable in size for proteomics experiments.

To the best of our knowledge, this is the first attempt to combine partial digestion of cell surface and primary amino group- labelling to produce more extracellular topology data for TMPs. These new experimental data can be used as extracellular constraints in the CCTOP algorithm to have a more accurate prediction. The refined TMP topology can be used as a starting point for modelling their 3D structures or designing laboratory experiments (e.g. revealing accessible regions for antibody or small molecule drug design).

## Results

### The developed method and the optimal pre-digestion conditions

**Figure 2:**
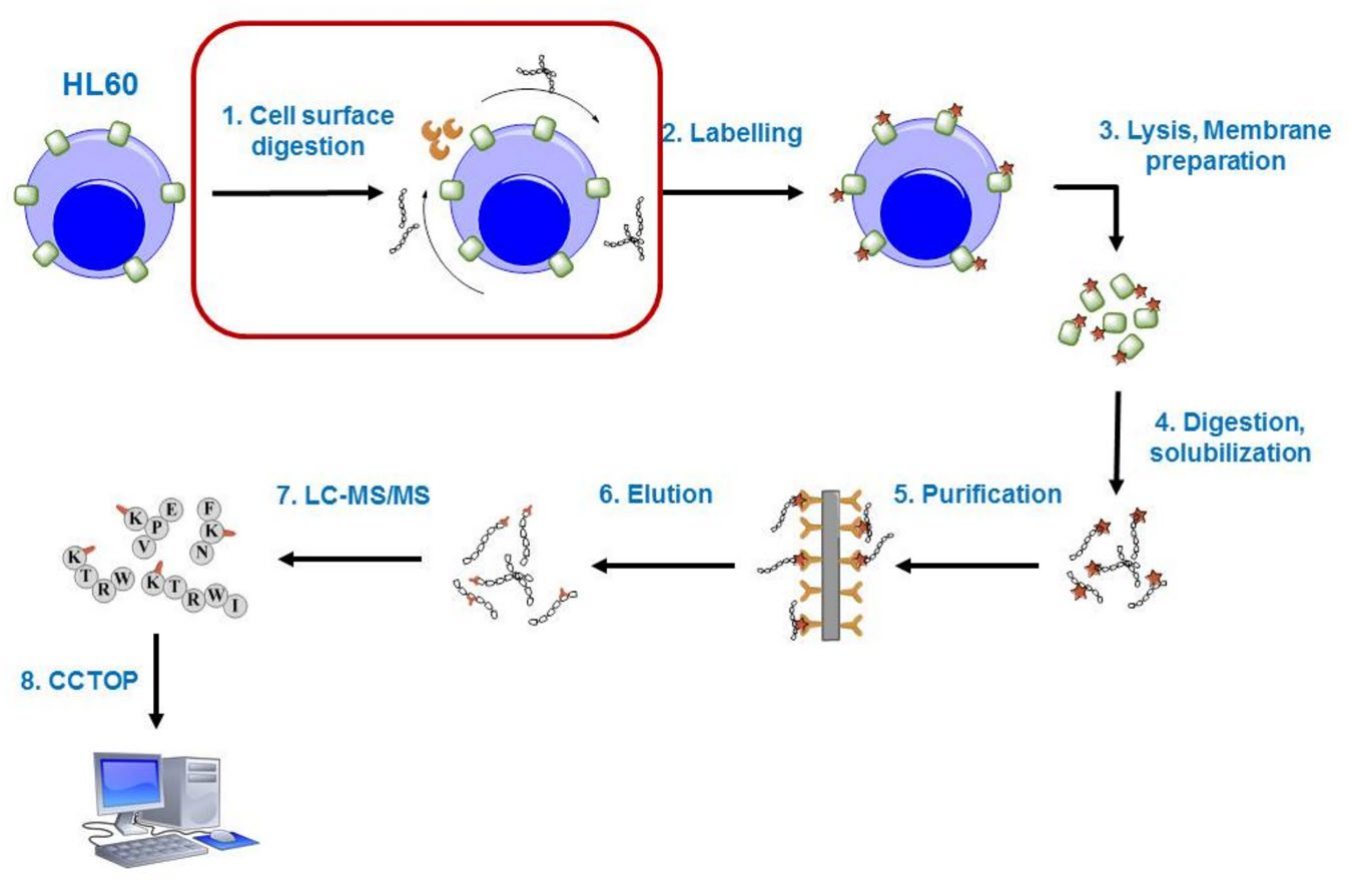
Flowchart of the new topology characterization method. Isolated cells are pre-digested by serine proteases (trypsin or chymotrypsin) and labelled with a membrane-impermeable, primary amino group-specific labelling agent (Sulfo-NHS-SS-biotin). The cells are lysed; plasma membranes are isolated, then solubilized and digested with proteomics-grade trypsin. The modified peptides are purified on a high-capacity neutravidin agarose resin, then eluted by reducing agent and sequenced by tandem mass spectrometry. Identified labelled positions can be used as extracellular constraints in the CCTOP topology prediction algorithm. The red rounded rectangle highlights the new step in the protocol compared to the previously published method^43^.

To overcome the limitations of the methods described above and to generate more topology data, we modified our previously published cell surface labelling technique. The flowchart of the developed method is depicted in Fig. 2, where the red rounded rectangle marks the main difference to our earlier protocol.

Two different serine proteases were used in the pre-digestion process (trypsin or chymotrypsin) in separate experiments. Digestion conditions were optimized to avoid cell damages, preventing intracellular labelling of TMPs. In the control experiments, trypsin or chymotrypsin were used in a range of 0.0039–1 mg/ml (two-fold dilution series) in PBS (pH  =  7.4) on equal numbers of HL60 cells and incubated for 5 min 37 °C (trypsin) or for 5 min 30 °C (chymotrypsin). We have determined the optimal concentration and digestion time of enzymes that had no/minimal effect on the integrity of the cells. The latter was monitored by flow cytometry, based on the uptake of propidium iodide dye (see details in the Supplementary Methods, Supplementary Results and Supplementary Figs. 1, 2). According to these data, we choose 0.0312 mg/ml for trypsin and 0.0078 mg/ml for chymotrypsin in the pre-digestion step. The cells were treated with these enzymes for two different time periods (trypsin: 15 min or 25 min, chymotrypsin: 10 min or 20 min). For both treatments, untreated cells were used as controls (marked by NPT: non-pre-digested by trypsin or NPC: non-pre-digested by chymotrypsin).

### Monitoring the cell surface labelling by confocal microscopy

**Figure 3:**
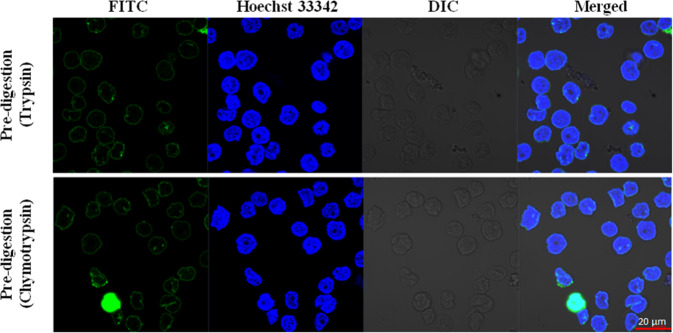
Sulfo-NHS-SS-biotin does not penetrate the plasma membranes of enzyme-treated cells. The cells were treated with the previously determined maximal tolerable trypsin or chymotrypsin concentrations, biotinylated and location of the dyes and cells were detected (from left to right: FITC conjugated anti-biotin antibody fluorescence, Hoechst 33342 DNA dye fluorescence, Differential Interference Contrast (DIC) and Merged picture). Red scale bar: 20 µm. Zeiss ZEN lite software (Carl Zeiss, Oberkochen, Germany) was used to acquire images.

Although the correct digestion conditions were optimized by flow cytometry experiments, we also wanted to confirm their accuracy with confocal microscopy. After partial proteolysis, 2 mM Sulfo-NHS-SS-biotin was used to label cell surfaces in the different samples. The efficiency of extracellular labelling was validated by confocal microscopy (Fig. 3). Based on the location of the fluorescein (FITC) fluorophore that was conjugated to the monoclonal anti-biotin antibody we can confirm that both trypsin- and chymotrypsin-treated HL60 cells were biotinylated mainly on the cell surface. There was no signal in the cytoplasm (except for one cell in the chymotrypsin-treated sample, where the whole cell glows green, Fig. 3, second row, FITC column) confirming that the used conditions do not result in internal labelling. The minimal cytoplasmic labelling does not disturb the whole process, due to the membrane preparations and several washing- and purification- steps, the inside labelled parts of the TMPs remain below the detection limit. Control samples are presented in Supplementary Fig. 3, where the cytosolic FITC fluorescence after the TritonX-100 permeabilization verifies that the labelling agent can penetrate the plasma membrane just after the permeabilization of the cell membranes.

### Membrane preparations and enrichment of labelled protein segments

Labelling of the pre-digested HL60 cells was followed by membrane isolation and the protein content of the samples was determined by the Lowry method. Membrane preparations were solubilized with 0.1% Rapigest, then pre-treated with PNGaseF and finally digested with proteomics-grade trypsin (see Methods). SDS-PAGE was used to test the reproducibility of the preparation process and the effect of pre-digestion for the protein pattern on the gel (Supplementary Fig. 4), and to verify the successful digestion before affinity isolation.

The biotinylated peptides were isolated on high-capacity neutravidin agarose and the aspecific non-labelled peptides were discarded from the column by hard washing steps. Different fractions of the affinity purification process were blotted onto a PVDF membrane to quantify the presence of the biotinylated peptides/components. The dot-blot method confirmed that enough amount of neutravidin agarose was used to bind all biotinylated components of each sample (Supplementary Fig. 5). This method also proved that we did not wash away a significant amount of labelled components.

Covalently biotinylated peptides were eluted with DTT reducing agent from the neutravidin agarose-filled column and alkylated by iodoacetamide. This step resulted in the covalent modification of extracellularly accessible primary amino groups (Table 1) either by +87.998 Da (Thioacyl modification, non-alkylated form) or +145.020 Da (CAMthiopropanoyl, alkylated form), as described in our earlier work^43^. These modifications could occur on protein N-termini, lysine side chains and peptide N-termini (due to the pre-digestion of the cell surface).

**Table 1:** Covalent modifications on extracellular primary amino groups.

Name	Mass shift (Da)	Target amino acid or N-terminal	Structure
Thioacyl	+87.9983	Lysine (K), Protein N-termini, Peptide N-termini	
CAMthiopropanoyl	+145.0198	Lysine (K), Protein N-termini, Peptide N-termini	

### Identification of the labelled peptides by tandem mass spectrometry

The eluted peptide mixes were purified on C18 spin columns, then peptides were analysed by nanoLC-MS/MS. Potential extracellular modifications, listed in Table 1, were searched using Byonic search engine (Supplementary Table 1/‘raw ms data’ sheet). Trypsin and chymotrypsin pre-digestion resulted in 4344 labelled peptides, 84% of them belonging to TMPs (Supplementary Table 1/Trypsin sheet) and 5594 modified peptides (78% of them are TMPs, Supplementary Table 1/Chymotrypsin sheet), respectively. Control unlabelled membrane preparations were also analysed (Supplementary Table 1/‘Control unlabelled’ sheet), but only one peptide (highlighted with red background in the table) was identified as modified at least three times, so we did not take unlabelled samples into account in the labelled samples during our later analyses (in other words, our results of labelled samples did not need to be normalized by the results of control samples).

### Processing of labelled sites of TMPs and validation of the results

Modified peptides from various searches were mapped to SwissProt Homo Sapiens protein sequences. TMPs were identified using the CCTOP algorithm^15^. Only those labelled sites of TMPs that were detected with at least three modified peptides in the given treatment were considered. Using this filter, we identified 40–50 labelled TMPs (with at least one modified residue) from HL60 cells after different pre-digestion conditions (Table 2 and Supplementary Table 2).

**Table 2:** The number of the labelled TMPs and their labelled positions under different pre-digestion conditions.

Pre-digestion enzyme	Pre-digestion time	Labelled TMPs	Labelled positions of TMPs
**Chymotrypsin**	- (NPC)	46	97
	10 min	43	105
	20 min	41	91
**Trypsin**	- (NPT)	47	115
	10 min	51	120
	20 min	50	110

The left column indicates the digestion times of the two different enzymes (NPC and NPT mark the non-pre-digested controls).

**Figure 4:**
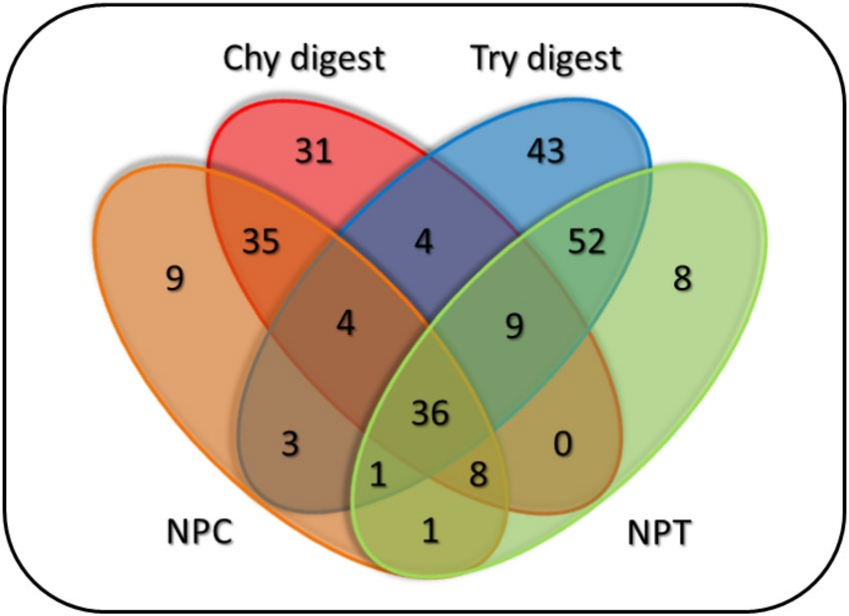
Venn diagram showing the number of individually labelled sites of TMPs from the HL60 cells, separated by enzymes and their treatment time. Chy digest and Try digest: merged chymotrypsin and trypsin pre-digested samples [10–20 min and 15–25 min, respectively]. NPC and NPT: control samples for chymotrypsin and trypsin enzyme treatments, respectively.

Effect of the pre-digestions to the number of the TMP’s labelled sites was evaluated by comparing the untreated samples (NPC and NPT, pre-incubated at different temperatures (30 °C or 37 °C) before the labelling process) and the merged results of treated samples of each enzyme (Fig. 4).

Figure 4 shows that the pre-digestion with different enzymes yielded new labelled sites for TMPs that were undetected in the control samples. Pre-digestion not only increased the number of newly found extracellular sites, but it also helped to detect new labelled TMPs (Supplementary Fig. 6). The labelled TMPs and sites of different experiments are presented for each enzyme on Supplementary Fig. 7.

To measure the accuracy of the modified labelling procedure, we compared the topological location of the labelled residues to the results of independent experiments that are listed in the TOPDB^42^. Previous experimental data confirms that the labelled positions are almost exclusively located in the extracellular regions (Table 3), proving our method almost 100% accurate, making it suitable for topology characterization. The longer pre-digestion time (25 min) has affected the correctness of topology data in the case of trypsin, indicating that the proteolysis time can be increased only up to a certain limit.

**Table 3:** The identified labelled sites were validated with other experimental topology data for the respective TMPs from TOPDB.

Localization in TOPDB	Chymotrypsin treatment			Trypsin treatment		
	NPC	10 min	20 min	NPT	15 min	25 min
Extracellular	65	73	65	86	87	79
Unknown	32	32	26	29	32	28
Intracellular	0	0	0	0	1	3

Header: protease for pre-digestion, first column: location of experimental data in the TOPDB. Extracellular: identified as extracellular in other experiments, Intracellular: not extracellular position in other experiments, Unknown: no data available.

## Discussion

Transmembrane proteins play important roles in cellular processes, their absence, malfunction or mutations may lead to the development of various diseases^48–51^. More accurate characterization of their structure is essential to understand their function, but the number of solved structures of TMPs to date is low, compared to their number in the different proteomes. Several alternative experimental and bioinformatics methods were developed to predict their topology, but all of them have limited accuracy.

The state-of-the-art topology prediction methods (like CCTOP) can use existing experimental results as constraints, making them more effective in the determination of the topology of TMPs, which leads to a more accurate prediction of their high-resolution structures as well. Existing experimental methods are not able to annotate many TMPs with at least one topologically characterized segment yet. Therefore, we would like to develop a method to produce more experimental data for some of the partly characterized and uncharacterized proteins.

In the present work, we introduce a new experimental topology characterization method that is based on our previously developed cell surface Sulfo-NHS-SS-biotin labelling technique. Here, we combined limited proteolysis of the cell surface with a primary amino group labelling method which together give rise to previously undetectable potential targets for the chemical modification on the cell surface. Two different serine proteases (trypsin and chymotrypsin) were utilized for the partial digestion of the HL60 cell surface. The optimal enzyme concentrations and digestion time periods were determined by flow cytometry experiments (Supplementary Figs. 1, 2), then these parameters were used for confocal microscopy experiments to verify that the labelling agent does not penetrate the plasma membrane (Fig. 3. and Supplementary Fig. 3). The labelled membranes were isolated, SDS-PAGE was used to test the reproducibility of the preparation process (Supplementary Fig. 4), and finally the samples were solubilized and digested. Using dot blot method, we also determined the sufficient amount of high-capacity neutravidin agarose required to bind the total biotin content of each pre-digested biotinylated membrane preparations (Supplementary Fig. 5). Most of the non-specific peptides were eliminated by adjusting the number of washing steps and various washing buffer compositions on the avidin resin. The latter is certified by the fact that ~97% of identified peptides are biotinylated or contain cysteine residues which can presumably remain on the affinity column via a disulphide bond with the labelled peptides (These cysteine-containing peptides are called ‘piggyback’ peptides by previous studies^43,52^. Of the almost 10,000 modified (+87.998 or +145.020 Da) peptides (Supplementary Table 1.), ~93% were identified with a + 145.020 Da modification that indicates that the alkylation step was successful.

The labelled peptides were mapped to the human proteome and by filtering for at least 3 peptides for each type of experiment (i.e. enzyme type and digestion time), altogether 223 extracellular positions were identified in 75 TMPs in HL60 cell line by 25 nanoLC-MS/MS runs (Supplementary Table 2).

164 out of 223 positions were characterized previously in the TOPDB database with 98.2% of the 164 labelled primary amino groups being located in the extracellular region in earlier experiments, therefore the data produced by our new procedure is near 100% accurate. Although we found a few controversial labelled positions in the 15 and 25 min trypsin pre-digested samples (one inside position from 15 min and three from 25 min pre-digested samples, Table 3), these indicate that the too long pre-digestion time may affect the quality of the produced topology data. Altogether 59 new extracellular positions were determined in 32 uncharacterized TMPs.

The essence of the new experimental method is the pre-digestion of the cells with the proteolytic enzymes such as trypsin and chymotrypsin prior to the Sulfo-NHS-SS-biotin labelling of the cell surface, giving rise to previously undetectable positions of TMPs that become available for the biotinylation reaction in either of two ways. First, 37% of all N-terminally labelled peptides was only detected in trypsin and chymotrypsin pre-digested samples, indicating the formation of previously non-existent N-terminals. Second, 30% of all modified sites originating from pre-digested samples implies that the proteases help to uncover once inaccessible regions of TMPs. However, some labelled sites were lost due to pre-digestion, i.e. identified in NPC and NPT samples only, most of them being located in large extracellular domains.

The bulk of the identified TMPs (56 out of 75 labelled TMPs) are Single-pass type I or II proteins, having only one TM segment and a large extracellular domain. Their extracellular domains contain many lysine residues or peptide N-termini (especially after the digestion) that became available for the biotinylation agents during pre-digestion.

Topology prediction of multi-pass TMPs can benefit even more of the presented protocol and we have identified 19 of such proteins. Their topology predictions are often controversial because their structures are more complex, e.g. membrane-embedded parts may contain different elements such as re-entrant loops or interfacial helixes, which could sometimes deceive the prediction algorithm. For example, in the case of the Neutral amino acid transporter B(0) protein (AAAT_HUMAN), different prediction algorithms provided various topologies^4^. However, the new method, presented here identified one labelled position (Ser-165) between the third and fourth TMSs (both in trypsin and chymotrypsin pre-digested samples) that prove the extracellular location of this loop region of the protein, which is consistent with the previously determined N-linked glycosylation site from this part of the protein^33^. The extracellular location of this loop has also been validated by our previous carboxyl group-labelling method resulting in three modified positions (Asp-173, Glu-209/210)^44^ as well as by primary amino group-labelling method (Lys-178)^43^. Additionally, the 3D structure of this protein was solved by cryo-electron microscopy (PDB code: 6GCT)^53^ suggesting that all modified positions are localised in the extracellular region, confirming the accuracy of our data. In the case of the leukocyte surface antigen CD47 protein (CD47_HUMAN), another similar multi-pass TMP, the location of the N-terminal region is predicted differentially^4^, but various existing topology data^33,54,55^ and labelled sites of this method verify that the N-terminal of this protein is located on the extracellular side.

By comparing the number of labelled peptides of the new method to that of our previous procedure^43^, we found that the earlier study resulted in 371 modified positions in 114 TMPs that is slightly more than the number of the currently detected ones. The difference can be explained by three reasons: i, in each experiment, cells were incubated at the optimal temperature of digestion for 10–25 min before labelling, allowing proteins to be internalized from the cell surface; ii, in the present work, 4–5 samples were made for each time period, while previous results originated from 12 parallel measurements on a Q-TOF, therefore ‘confirm modified position with three peptides’ filter could yield more data; iii, in the present study, the labelled peptides were searched against the SwissProt database, while previously the search database was the combined SwissProt-TrEMBL database and the size of search space may affect the peptide identification^56^. Although only 75 labelled TMPs were identified here and 56 out of these TMPs show homology with our previous results, our new protocol determined topology data for 19 other TMPs that were not characterized in the original method. Additionally, the specificity of our newly presented method grew for modified segments of TMPs (~75–80% labelled peptides originated from TMPs, compared to the previous ~50%) due to the pre-digestion step and the several applied washing steps. We also found less conflicting positions in these experiments as compared to the existing experiments from the TOPDB. Considering the modified nonTM proteins we got labelled peptides from peripheral membrane proteins, which indicates that our protocol can detect these proteins on the extracellular surface of the cells as well.

To summarise, the advantage of the limited-proteolysis combined Sulfo-NHS-SS-biotin labelling method is that by providing reliable topology data for many TMPs it contributes to their more accurate topology predictions. The time required for the experiment from starting cell culturing to getting the results of tandem mass spectrometry was about 3–4 weeks which is faster than the majority of the previously developed topology data gathering methods, that sometimes characterize only a single protein as described in the introduction.

Although the new method resulted in many new extracellularly labelled sites for TMPs, there is still room for improvement. We can apply multi-enzyme digestions (both during the pre-digestion of the cell surface and the digestion of membrane preparations) which might give rise to a broader range of identified proteins per sample^57^. Furthermore, applying the improved method to other cell lines or their isolated organelles could identify specific accessible segments of proteins that can be important for the design of active compounds. Finally, the cell surface biotinylation techniques combined with different limited proteolysis steps could discover new potential targets from the given cell surface.

## Methods

### Cell culture

HL60 (ATCC CCL-240) – a suspension acute promyelocytic leukemia cell line - cells were used in each experiment. The cells were maintained under sterile conditions and grown in RPMI medium (Gibco, RPMI 1640 Medium Glutamax Supplement HEPES, Thermo Scientific) supplemented with 10% Fetal Bovin Serum (FBS, Gibco, Thermo Scientific) and 50 µg/ml Penicillin-Streptomycin (Gibco, Thermo Scientific). All completed media were sterilized by bottle-top vacuum filter systems (Corning, 0.22 µm pore size, Sigma-Aldrich). Every 2–3 days, cells were passaged under a laminar box (ESCO Class II Bsc) and incubated in T25 or T75 flasks (Eppendorf) in a humidified atmosphere containing 5% CO2 at 37 °C (Eppendorf, Galaxy 170 R). Depending on the size of the cultured cells, 3–4*10^7 HL60 cells were used for each proteomic assay.

### Cell isolation

HL60 cells were isolated by centrifugation at 300 g for 3 min at 4 °C, the medium and cells were gently resuspended with cold (4 °C) Phosphate-buffered saline (PBS; 137 mM NaCl, 2.7 mM KCl, 10 mM Na_2_HPO_4_ and 1.8 mM KH_2_PO_4_; pH=7.4, all component were purchased from Sigma-Aldrich) and centrifuged the same way again. The washing step was repeated twice, and in the last step was supplemented with an alkylating agent (4 mM iodoacetamide, Sigma-Aldrich) and incubated for 20 min in dark for blocking free sulfhydryl groups, as described in our previous works^43,44^.

### Partial proteolysis of the proteins on the surface of living cell

The HL60 cells were subjected to partial proteolysis before primary amine labelling by Sulfo-NHS-SS-biotin (Thermo Scientific). Enzymatic treatment was performed with two different enzymes: trypsin from porcine pancreas (Sigma-Aldrich) that cleave the C-terminal of the arginine and lysine residues and α-Chymotrypsin (Sigma-Aldrich) from the bovine pancreas which have more cleavage sites such as C-terminal of phenylalanine, tyrosine, tryptophan, methionine and leucine residues. The optimal enzyme concentrations and digestion times were determined by TC20 automated cell counter (Bio-Rad) or FACS Attune Acoustic Focusing Cytometer (Applied Biosystems). To determine the appropriate concentrations, a 1:2 serial dilution was made with PBS from 1 mg/ml stock solution of each enzyme. The optimal treatment time was analysed the same way, and the integrity of the cells was analysed by propidium iodide uptake (for details see in Supplementary Methods and Results). The proper enzyme concentrations (trypsin: 0.03125 mg/ml, chymotrypsin: 0.0078 mg/ml) and different digestion times (trypsin: non-pre-digested with trypsin (NPT), 15 min and 25 min; chymotrypsin: non-pre-digested with chymotrypsin (NPC), 10 min and 20 min) were used at optimal temperature (trypsin: 37 °C, chymotrypsin: 30 °C) and the proteolysis was stopped by adding cold 1% BSA in PBS (m/V) before each labelling experiment. In details, 2–2 ml final reaction volumes were applied for each cell surface digestion experiment (containing the cells and the appropriate amount of enzymes at the optimal temperature and buffer composition). After digestion, 8–8 ml cold (4 °C) 1% BSA in PBS were added immediately to each sample (thus, the temperature of the solutions decreased below the optimal temperature of the used enzymes and the supplemented BSA was an alternative substrate for the active proteases). Immediately after adding the BSA, the pre-digested HL60 cells were pelleted by centrifugation (300 g for 3 min at 4 °C) and supernatants were completely removed (thus, the proteases were also removed). Before the labelling process, the cells were washed with 10–10 ml cold PBS and centrifuged again with similar conditions (300 g for 3 min at 4 °C). Then the supernatants were removed again (further reducing the trace of the enzymes) and the cells were kept on ice before the biotinylation step.

### Labelling the amino groups on the surface of living cells

The commercially available Sulfo-NHS-SS-biotin (Thermo Scientific) membrane- impermeable agent was used to label the primary amine groups of accessible residues after the partial proteolysis. The enzyme treated or not treated (NPC or NPT) HL60 cells were incubated with this chemical reagent, the reaction was performed as described in our previous work^43^, the cells were labelled with ~2 mM Sulfo-NHS-SS-biotin in isotonic solution (PBS, to prevent cell rupture) at 4 °C for 20 min. The biotinylation process was stopped by adding 25 mM Tris buffered saline (TBS, 25 mM Tris, 150 mM NaCl; pH=7.4, Thermo Scientific). The labelled cells were pelleted by centrifugation (300 g for 3 min at 4 °C), the excess of labelling agent was removed by two TBS washing steps. Successful labelling of cell surface and membrane impermeability of labelling agent were examined by confocal microscopy (Supplementary Method).

### Membrane preparation

The abundance of the TMPs is significantly different from the cytosolic proteins in the cells^43,58^, therefore a membrane preparation strategy was used to facilitate the peptide identification from TMPs by mass spectrometry. Lysis of the HL60 cells were achieved by mechanical actions in Hypotonic lysis buffer (20 mM Tris-HCl, 10 mM KCl, 20 mM sucrose; pH = 7.4) that was supplemented with 10 mM iodoacetamide. Mechanical disruption of HL60 cells were performed on ice by micro-pestle (40 times, Sigma-Aldrich) and the solutions were passed through a 26-gauge needle (20 times, Sigma-Aldrich) with a disposable 1 ml syringes. The homogenates were centrifuged at 1700 g for 7 min at 4 °C for separating the intact cells, nuclei and cell debris. Subsequently, samples were transferred to a 10.4 ml polycarbonate tube (Beckman) and centrifuged at 100,000 g for 1 hour at 4 °C for using a 70.1 Ti fix rotor (Beckman) in L7–55 ultracentrifuge (Beckman). After ultracentrifugation, the membrane pellet was localized at the bottom of the tube, the supernatant was discarded and the pellet was washed once with 10 times diluted lysis buffer (pH = 7.7), finally centrifuged again at 100,000 g for 1 hour at 4 °C. The pellet was resuspended in the diluted lysis buffer and homogenized by Dounce tissue grinder pestle (2 ml, Sigma-Aldrich). Protein concentration of the preparations was measured by method of Lowry *et al*.^59^. We used SDS-PAGE to test the reproducibility of the preparation process, using HL60 samples produced at different times, but with the same protein content (Supplementary Method).

### Solubilisation and digestion of membrane preparations

The used protocol was similar to our previous work^43^. Membrane preparations (these average of protein content was ~300 µg/sample) were solubilized in 50 mM ammonium-bicarbonate buffer (NH_4_HCO_3_; pH=8, Sigma-Aldrich) in the presence of 0.1% (w/V) Rapigest detergent (Waters), 1 mM iodoacetamid and 1 mM BHES (Sigma-Aldrich). Surfactant solubilisation was assisted by 5*1 min sonication (Elmasonic S 30 H, Elma) at 4 °C followed by incubation on ice for 30 min (vortex every 5 min for improved solubilisation). The solubilised mixture was treated with PNGaseF (500 unit/sample, New England Biolabs) for 2 hours at 37 °C, then proteomics grade trypsin (Sigma-Aldrich) was added in a 1:50 ratio (enzyme: protein; w-w) and incubated for 16 hours at 37 °C. At the end of digestion, the reaction was stopped by heat inactivation for 10 min at 95 °C and by TLCK hydrochloride trypsin inhibitor for 20 min at room temperature (100 µM Sigma-Aldrich). Digestion efficiencies were monitored SDS-PAGE too (Supplementary Methods).

### Biotinylated peptide isolation on neutravidin agarose

Sulfo-NHS-SS-biotin labelled peptides were immobilized on high capacity neutravidin agarose (Thermo Scientific). The binding efficiencies of the column were examined by dot-blot experiments (Supplementary Methods), in order to determine the optimal volume of neutravidin agarose (100 µl/sample). Labelled mixtures were incubated on affinity columns for 1 hour at room temperature. Columns were washed extensively to reduce the number of non-specific peptides or contaminants as described by Lango *et al*.^43^. The covalently modified peptides were eluted from the column with 50 mM DTT in 50 mM NH_4_HCO_3_ (pH = 8) for 1 hour at 37 °C, which were followed by an alkylation step with 110 mM iodoacetamide in dark at room temperature.

### Isolated peptide identification by mass spectrometry

Before mass spectrometry analysis the peptide mixtures were dried using a SpeedVac concentrator and were purified on C18 spin column (Thermo Scientific) according to manufacturer’s instructions. The samples were dried again then diluted in 20 µl loading buffer containing 2% acetonitrile and 0.1% formic acid. 6–6 µl was injected and analysed by nanoLC-MS/MS similarly to our previous work^43^. The used mass spectrometer was a Maxis II QTOF (Bruker Daltonics) with CaptiveSpray nanoBooster ionization source coupled to a nanoLC (Dionex Ultimate 3000 NanoLC System, Sunnyvale). The peptides were trapped on Acclaim PepMap100 C18 Nano-Trap column (5 μm, 100 Å, 100 μm × 20 mm, Thermo Scientific) and separated online using an Acquity UPLC M-Class Peptide BEH C18 column (25 cm, 1.7 μm particle size, Waters). A gradient elution with two different solvents was applied (A: water + 0,1% (V/V) formic acid (FA); B: acetonitrile + 0,1% FA) for 100 min (first 2,5–25% solvent B in 80 min then 25–45% solvent B in 20 min). The cycle time of the MS measurements was set to 2.5 sec and MS spectra were acquired at 3 Hz in the 150–2200 m/z mass range while CID was performed at 4 or 16 Hz depending on the intensity of the precursor. The resulted raw data were recalibrated with Compass DataAnalysis software 4.3 (Bruker Daltonics) and MS/MS peak list was generated using ProteinScape software 3.1 (Bruker Daltonics). The labelled peptides were identified by Byonic 2.15.7 software, search engine parameters are presented in the Supplementary Table 1.

### Processing of the MS results

The results of the mass spectrometry runs were filtered for LogProb > =2, resulting in FDR < = 1%. The peptides carrying the artificial modifications by our labelling process (+87.998 Da or +145.020 Da) were filtered and listed in Supplementary Table 1.

The modified peptides were mapped to the human proteome (SwissProt) by blastp-short (with the options E-value: 1e6, wordsize: 6, threshold: 1e-7) in order to identify all proteins that contain similar peptides. We filtered for proteins with at least one modified position identified at least three times in each digestion time period. TMPs were identified by the CCTOP algorithm, and the topological location of their labelled position was evaluated based on earlier experimental results from TOPDB database. These labelled positions of TMPs and their topological validation (‘Extracellular’: the position was extracellular in at least one former experiment; ‘Intracellular’: the position was intracellular in the earlier experiments; ‘Unknown’: ‘no data available’) are listed in the Supplementary Table 2 separately for each enzyme and time period.

## Supplementary information

Supplementary information.

Supplementary Table 1

Supplementary Table 2
