# Supplementary material for: Partial proteolysis improves the identification of the extracellular segments of transmembrane proteins by surface biotinylation
Source: Sci Rep. 2020 Jun 1;10:8880. doi: 10.1038/s41598-020-65831-2 (PMC7264363; doi:10.1038/s41598-020-65831-2)
Supplement: Supplementary file 1 — Supplementary information. [file 41598_2020_65831_MOESM1_ESM.pdf]

Supplementary information for

Partial proteolysis improves the identification of the extracellular segments of transmembrane proteins by surface biotinylation

Tamás Langó[1], Zoltán Gergő Pataki[1,2], Lilla Turiák[3], András Ács[3], Julia Kornélia Varga[1], György Várady[1], Nóra Kucsma[1], László Drahos[3] & Gábor E. Tusnády[1]*

[1] Institute of Enzymology, Research Centre for Natural Sciences, Magyar tudósok krt 2, Budapest, H-1117 Hungary.

[2] Soft Flow Ltd. Ürögi fasor 2/a, Pécs, H-7634 Hungary

[3] Institute of Organic Chemistry, Research Centre for Natural Sciences, Magyar tudósok krt 2, Budapest, H-1117 Hungary.

*Correspondence and requests for materials should be addressed to G.E.T. (email: tusnady.gabor@ttk.hu)

Supplementary Methods

Optimization of cell surface digestion with flow cytometry

HL60 cells were treated with trypsin or chymotrypsin in a range of 0.0039-1 mg/ml (two-fold dilution series) in PBS (pH = 7.4) and incubated for 5min 37°C (trypsin) or for 5min 30°C (chymotrypsin). The prepared 9 different concentrations of enzymes were added to an equal number of cells which treated at 30°C (chymotrypsin digestion) or at 37°C (trypsin digestion) for 5 min. After the treatment time, the reactions were stopped with cold 1 % (m/V) BSA (Sigma-Aldrich) in PBS solutions and the cells were pelleted by centrifugation. The cells were stained with 1000x diluted propidium iodide (the positive cells are dead, Thermo Scientific) in FACS experiments (Supplementary Figure 1.) or trypan blue (Gibco) in TC20 measurements (data not shown).

Similar numbers of HL60 cells were treated with the previously determined enzyme concentrations for different time periods (5, 10, 15, 20, 30, 45 and 60 min) at 37°C (or at 30°C at the chymotrypsin) in a water bath. The reaction was stopped and cells were stained as described above. Dead cell ratios were also determined based on propidium iodide uptake

(Supplementary Figure 2). Measurements of these and cell concentrations were conducted by FACS Attune Acoustic Focusing Cytometer.

Experiments by confocal microscopy

After the protease treatment, we used confocal microscopy to verify that the labelling was done on the appropriate part of the cell. The cells were pre-digested with the optimal enzyme concentration for the longest determined treatment times (trypsin: 25 min; chymotrypsin: 20 min) and labelled with 2 mM Sulfo-NHS-SS-biotin for 20 min at 4°C. The reaction was stopped as described in the Method section, and the cells were fixed with 4% paraformaldehyde for 5 min at 4°C, then permeabilized by 0,1% TritonX-100 for 2 min at room temperature. Thereafter, the cells were incubated with cold PBS containing 2% (w/v %) BSA for 40 min at 4°Cbefore applying FITC conjugated anti-biotin antibody (diluted 250x in 2% BSA-PBS, incubated for 40 min at 4°C) and propidium iodide DNA dye (diluted 1000x in PBS, 0.1 μg/ml, incubated for 5 min at 4°C). The cells were washed once with PBS, mounted to the microscope slides and location of the dyes were analysed with a Zeiss LSCM 710 microscope using a 63x NA=1.4 Plan Apo objective (Figures 3). Positive control samples (reflecting the cytosolic labelling) were produced on the same way, except for labelling them with biotinylation agent after the permeabilization. Two positive samples were produced: first non-labelled cells were permeabilized with TritonX-100 and biotinylated. The second positive sample started with labelled cells which were permeabilized and labelled again. Negative controls were prepared the same way, without any kind of labelling agents. Results of control samples can be seen in the Supplementary Figure 3.

SDS-PAGE

After the different pre-digestion and the successful biotinylation the cells were lysed and membranes were isolated. The reproducibility of preparations and the effect of pre-digestion on the membrane preps were examined with SDS-PAGE method (Supplementary Figure 4). The samples were boiled at 95°C for 5 min in the presence of DTT containing SDS loading buffer, and separated on polyacrylamide gel (4% stacking gel and 12% separation gel). Sharpmass VII (Euroclone) marker was used for mass identification. Stacking was performed for 10 min at 100V, then the separation for 60-90 min at 140V. Thereafter, the gel was incubated with Coomassie BBR-250 at a concentration of 2 mg/ml for overnight. The separated proteins were detected by ChemiDoc XRS+ (Bio-Rad).

Dot-blot

The biotinylated membrane preparations were digested, then biotinylated components were purified on the neutravidin agarose-filled column. The biotin content of each fraction before, during and after the affinity isolation was determined by dot-blot method. These fractions were diluted 100-fold and were blotted onto a 0.45 μm PVDF membrane using a Bio-Dot microfiltration apparatus (Bio-Rad). PVDF membranes were incubated in blocking buffer (25 mM TRIS.HCl, pH=7.4, 2.7 mM KCl, 137 mM NaCl, 0.05% Tween-20) containing 2% BSA for 30 min at room temperature. Thereafter HRP conjugated avidin was diluted 50000-fold in the blocking buffer and the membrane was incubated similarly to the previous step. For the visualization of the labelled components the enhanced chemiluminescence reagent (Merck) was applied. The images were captured by a ChemiDoc XRS+ Imaging system (Bio-Rad, Supplementary Figure 5).

Supplementary Results

Results of flow cytometry experiment

Optimal enzyme concentrations and digestion times were determined by flow cytometry as described in the Supplementary Methods. Supplementary Figure 1 depicts the change of dead cell ratio vs the applied enzyme concentrations for the trypsin. The green line indicates the concentration limit that cells can bear without aggregation (indicating the integrity of the cells) and the green arrows denote the selected concentrations (0.03125 mg/ml and 0.0078 mg/ml for trypsin and chymotrypsin, respectively) for the pre-digestion protocol.

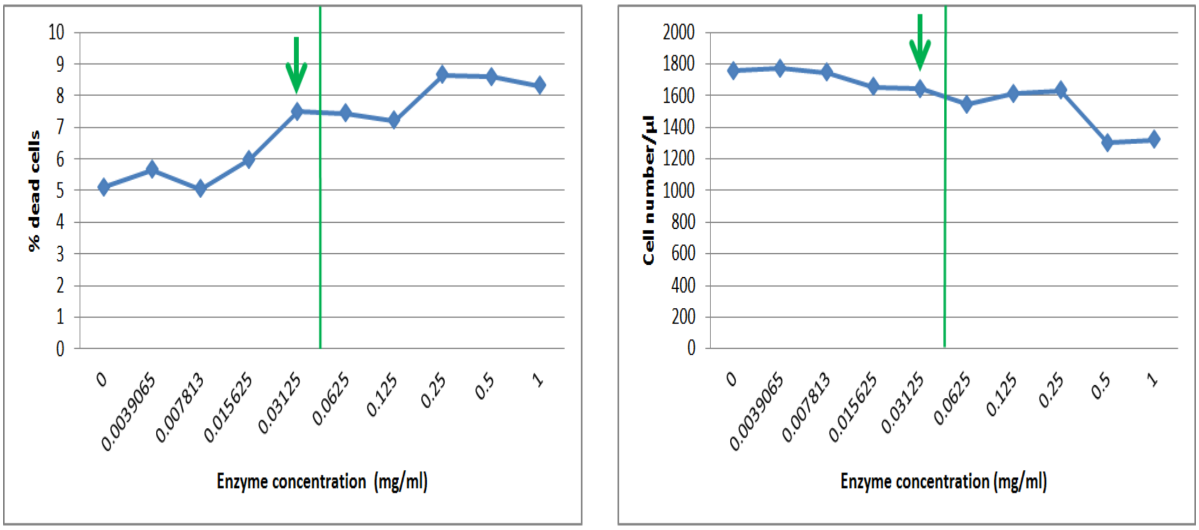

Supplementary Figure 1. Determination of the optimal enzyme concentration.

Proteases were used in various concentrations for cell surface pre-digestion of HL60 cells. Change of dead cell rate and cell concentration were monitored. The green vertical lines mark the concentrations where aggregated cells started to appear. The green arrows mark the selected concentrations.

Optimal digestion time was determined in a similar manner. Death cell rate and cell concentration for different times were monitored for each sample (Supplementary Figure 2). Digestion times with a minimal effect on the integrity of HL60 cells were selected (15 and 25 min for trypsin, 10 and 20 min for chymotrypsin).

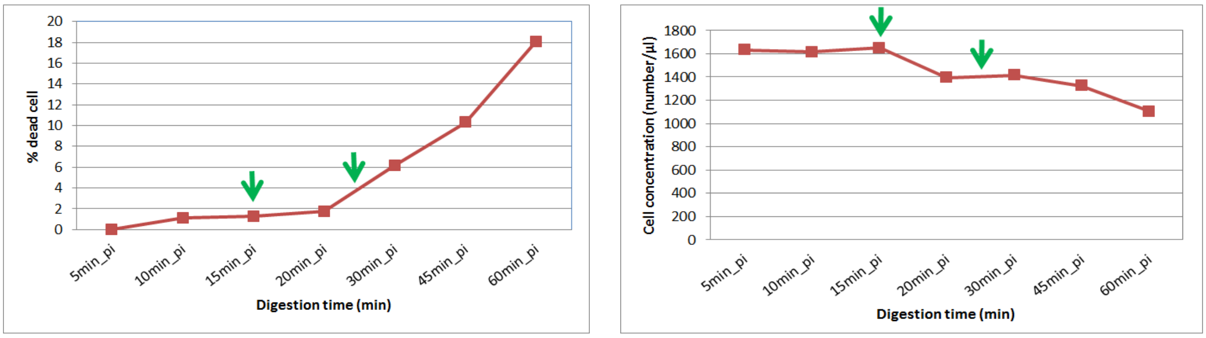

Supplementary Figure 2. Determination of the optimal digestion time.

Determined trypsin concentrations were used for various time periods for cell surface pre-digestion of HL60 cells. The integrity of the cells were monitored by the change of dead cell rate and cell concentration with FACS measurements. The green arrows mark two different time periods for trypsin that were chosen for the pre-digestion protocols.

Characterization control samples of cell surface labelling by confocal microscopy

After HL60 cells were pre-digested with the adjusted concentration of enzymes (trypsin and chymotrypsin) for the appropriate times, we examined the effect of longest digestion times on the permeability of plasma membranes. Although we got a homogenous FITC fluorescence on the cell surface (Figure 3), we wanted to verify its validity with the appropriate positive and negative controls (such as cytosolic/inside labelled positive controls and non-labelled negative controls, Supplementary Figure 3).

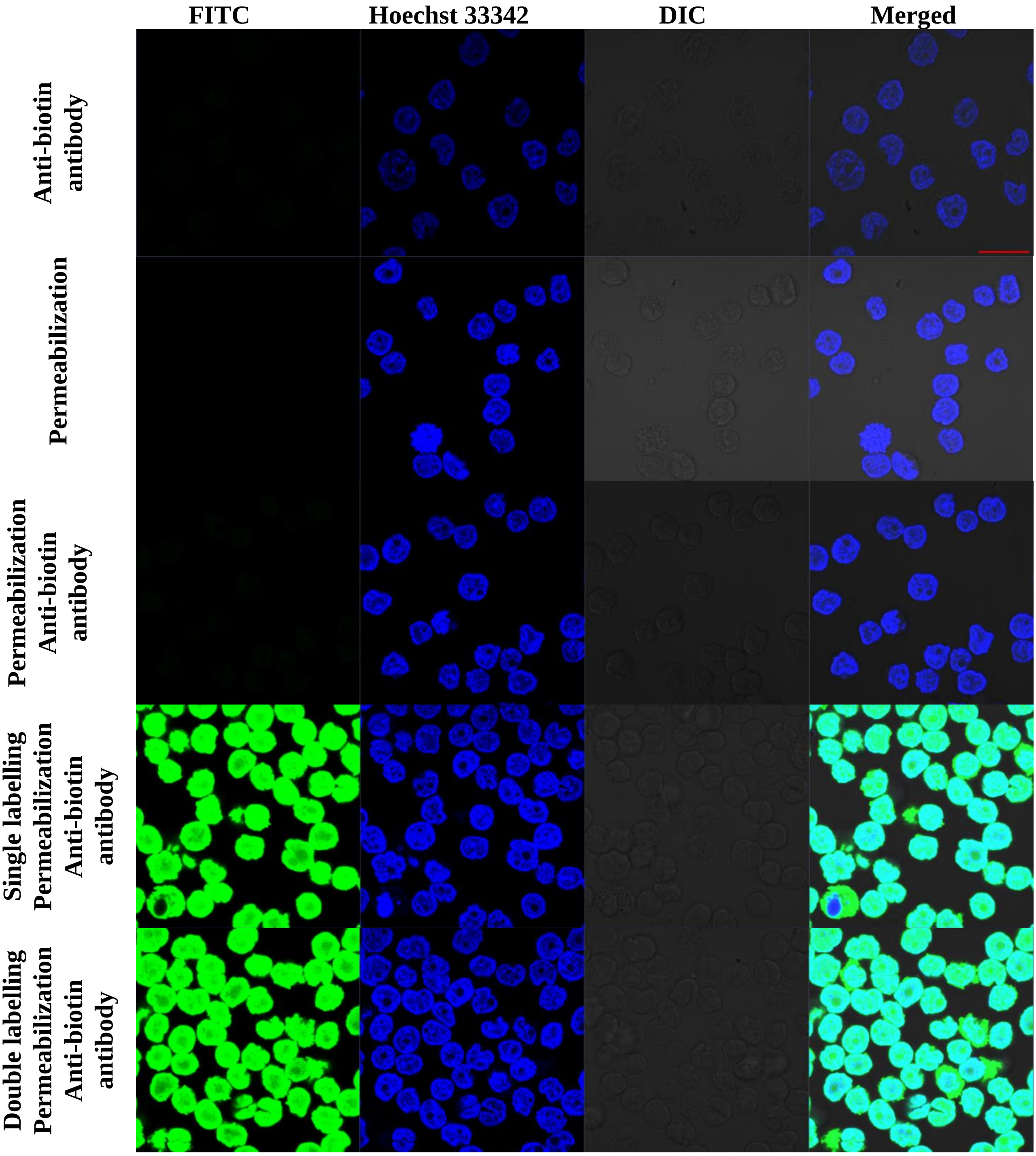

Supplementary Figure 3. Positive and negative control experiments for surface protein biotinylation of HL60 cells after pre-digestion treatment.

The first lane shows the target of the measurement (from left to right: FITC conjugated anti-biotin antibody fluorescence, Hoechst 33342 DNA dye fluorescence, Differential Interference Contrast (DIC) and Merged picture) and the first column indicates the applied treatments for each HL60 (TritonX-100 for permeabilization, FITC conjugated anti-biotin antibody for

detection, and Sulfo-NHS-SS-biotin as a labelling agent). The images were created by Zeiss ZEN lite software (Carl Zeiss, Oberkochen, Germany), the length of the red scale bar is 20 µm in the upper right corner.

Gel electrophoresis

SDS-PAGE was used to determine the reproducibility of membrane preparation (details in the Supplementary Methods). Similar protein patterns were observed for different trypsin pre-digested samples (Supplementary Figure 4, labels above the lanes indicate the different pre-digestion times: NPT, 15 or 25 min and '+S' stands for sonication) that indicate the preparation process is reproducible and partial cell surface digestions do not affect the protein pattern.

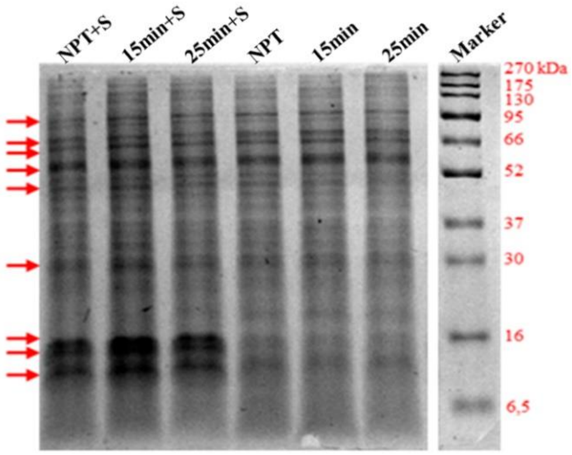

Supplementary Figure 4. Protein patterns of the membrane preparations of trypsin pre-digested samples.

The pre-digestion times are indicated above: NPT (non-pre-digested), 15min and 25min. Lysis was helped with sonication for some samples (labelled '+S'). The Coomassie-stained proteins and the marker lane were detected by a ChemiDoc XRS+ system. Red arrows indicate the most abundant protein bands that were consistently detected for each sample (indicating the reproducibility).

Results of dot blot experiment

Results presented in Supplementary Figure 5 show that the binding capacity of the used neutravidin agarose did not limit the enrichment of the biotinylated peptides. Biotinylated components were verified to remain bound to the neutravidin loaded columns as biotin was not detected in the flow-through fractions.

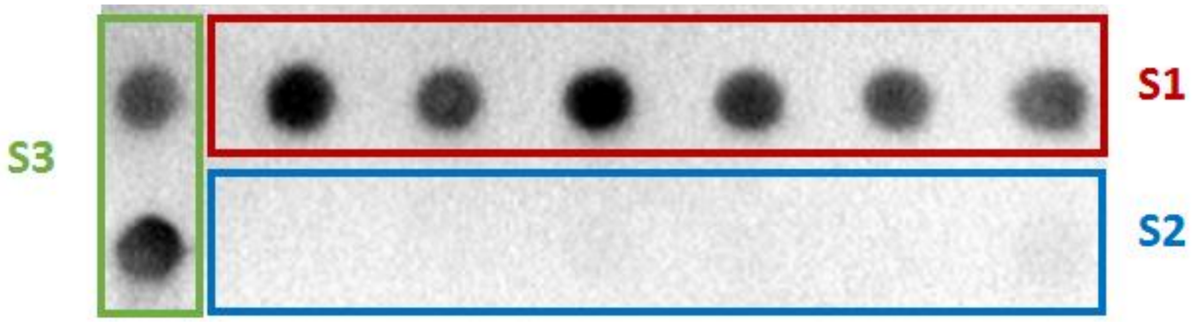

Supplementary Figure 5. Dot blot analysis of the primary amino group labelled HL60 samples.

Biotin content of different pre-digested membrane preparations and their fractions around the affinity chromatography were analysed with dot-blot method. S1: primary amino group labelled samples before affinity isolation, S2: flow through fractions of biotin-labelled samples after the biotinylated components were precipitated on affinity column, S3: biotinylated positive controls. Biotin content-dependent chemiluminescence was detected by a ChemiDoc XRS+ Imaging system.

Identified labelled TMPs and their labelled sites by tandem mass spectrometry

The list of the modified peptides is presented in the Supplementary Table 1. These peptides were mapped to the human protein sequences (SwissProt) with blastp and those sites that were identified at least three times in the given pre-digestion time period and belonged to TMPs, were gathered (These are presented in Supplementary Table 2, where the sheets are named after the pre-digestion time periods and enzymes). Several comparisons have been made that are presented in Venn diagrams (Supplementary Figure 6-7).

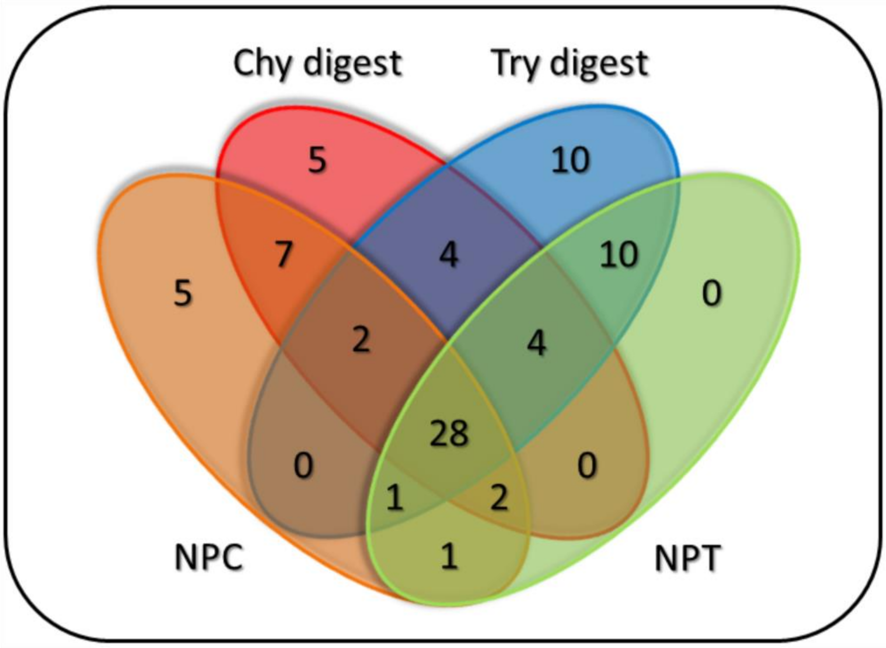

Supplementary Figure 6. Venn diagram depicting the number of individually labelled TMPs separated based on enzymes and treatment times (the marks are identical to Figure 4).

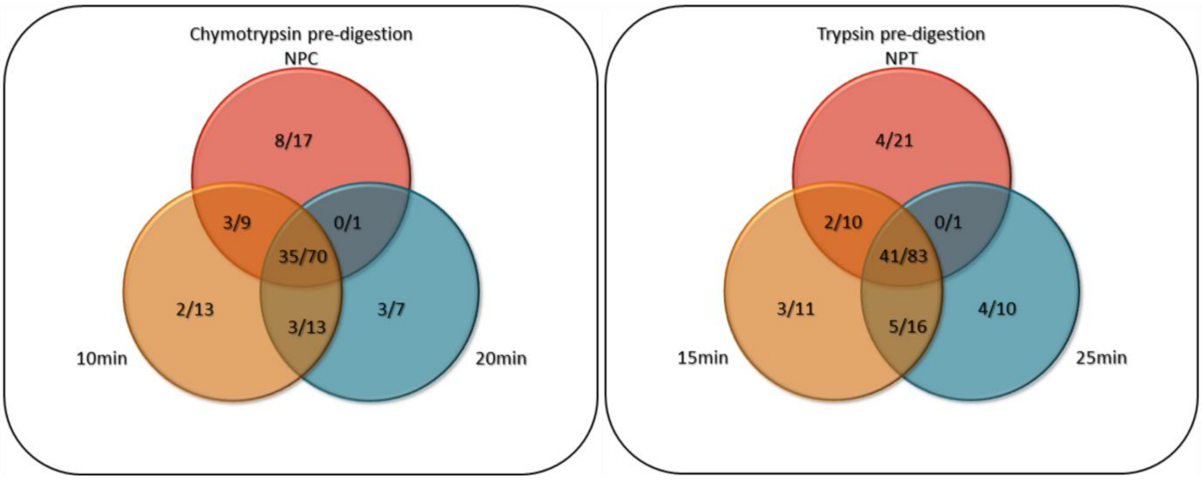

Supplementary Figure 7. Venn diagram showing the number of individually labelled TMPs/labelled positions, separated based on applied pre-digestion enzymes (left: chymotrypsin, right: trypsin) and treatment times.

Supplementary Tables

Supplementary Table 1. Byonic search engine parameters and list of the labelled peptides for each enzyme and control sample from HL60 cell line

see Supplementary_Table_1.xlsx

Supplementary Table 2. List of the labelled positions of TMPs and their topological validation

see Supplementary_Table_2.xlsx
